# Contrasting benefits of different artemisinin combination therapies as first-line malaria treatments using model-based cost-effectiveness analysis

**DOI:** 10.1038/ncomms6606

**Published:** 2014-11-26

**Authors:** Lucy C. Okell, Matthew Cairns, Jamie T. Griffin, Neil M. Ferguson, Joel Tarning, George Jagoe, Pierre Hugo, Mark Baker, Umberto D’Alessandro, Teun Bousema, David Ubben, Azra C. Ghani

**Affiliations:** 1 MRC Centre for Outbreak Analysis and Modelling, Department of Infectious Disease Epidemiology, Imperial College, London W2 1PG, UK; 2 MRC Tropical Epidemiology Group, London School of Hygiene and Tropical Medicine, London WC1E 7HT, UK; 3 Centre for Tropical Medicine, Nuffield Department of Medicine, University of Oxford, Oxford OX3 7LJ, UK; 4 Mahidol-Oxford Tropical Medicine Research Unit, Faculty of Tropical Medicine, Mahidol University, Nakhon Pathom 73170, Bangkok, Thailand; 5 Medicines for Malaria Venture, 1215 Geneva 15, Switzerland; 6 Prince Leopold Institute of Tropical Medicine, Department of Public Health, 2000 Antwerp, Belgium; 7 Medical Research Council Unit, PO Box 273 Banjul Fajara, The Gambia; 8 Faculty of Infectious and Tropical Diseases, London School of Hygiene and Tropical Medicine, London WC1E 7HT, UK; 9 Department of Medical Microbiology, Radboud University Medical Centre, 6525 GA Nijmegen, The Netherlands

## Abstract

There are currently several recommended drug regimens for uncomplicated *falciparum* malaria in Africa. Each has different properties that determine its impact on disease burden. Two major antimalarial policy options are artemether–lumefantrine (AL) and dihydroartemisinin–piperaquine (DHA–PQP). Clinical trial data show that DHA–PQP provides longer protection against reinfection, while AL is better at reducing patient infectiousness. Here we incorporate pharmacokinetic-pharmacodynamic factors, transmission-reducing effects and cost into a mathematical model and simulate malaria transmission and treatment in Africa, using geographically explicit data on transmission intensity and seasonality, population density, treatment access and outpatient costs. DHA–PQP has a modestly higher estimated impact than AL in 64% of the population at risk. Given current higher cost estimates for DHA–PQP, there is a slightly greater cost per case averted, except in areas with high, seasonally varying transmission where the impact is particularly large. We find that a locally optimized treatment policy can be highly cost effective for reducing clinical malaria burden.

## Abstract (web summary)

Several drug combinations with different properties are used for malaria treatment. Here, Okell *et al*. use a mathematical model to simulate malaria transmission and treatment with two drug combinations in Africa, and find that locally optimized policies can be highly cost effective for reducing malaria burden.

## Graphical abstract

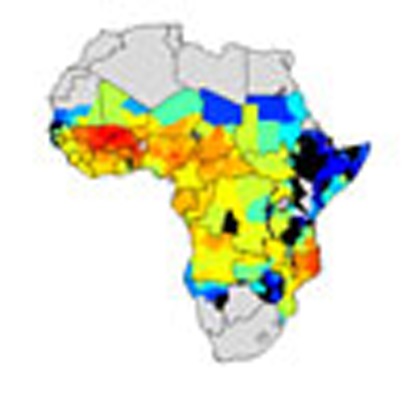

Artemisinin-based combination therapies (ACT) are the first-line-recommended treatments for uncomplicated *Plasmodium falciparum* malaria across nearly all malaria-endemic countries. The artemisinin derivative in the combination rapidly kills parasites but has a short half-life, while a partner drug with a longer half-life is given to clear remaining parasites after the artemisinin is no longer present. Five ACTs are currently recommended by the World Health Organization^1^: artemether–lumefantrine (AL), artesunate–amodiaquine (AS–AQ), artesunate–mefloquine (AS–MQ), artesunate–sulfadoxine–pyrimethamine (AS–SP) and, more recently, dihydroartemisinin–piperaquine (DHA–PQP). While the majority of countries recommend a single first-line treatment, several countries have either introduced or are considering multiple first-line therapies^2^. To date, choice of therapy has been based predominantly on cure rates for individuals (including assessment of existing drug resistance) and cost. However, now that many countries are aiming to substantially reduce malaria burden, the ability of the drug to reduce transmission is increasingly relevant^3^. Two properties of antimalarials can impact on transmission—(a) their ability to reduce onward transmission by rapidly killing circulating parasites and (b) the length of time for which the drug reduces the chance of reinfection. Understanding how these properties translate into impact and cost effectiveness can help countries choose optimal treatments for local populations.

Antimalarial drugs with long half-lives continue to benefit the patient after cure is achieved through post-treatment prophylaxis; that is, by protecting them against reinfection while the drug remains in the bloodstream. In areas with high malaria transmission and hence frequent re-exposure, this may be particularly important and could reduce malaria transmission within the whole community as well as protect individual patients, since preventing new infections also prevents future transmission^4^,^5^. Antimalarials that reduce a patient’s infectiousness likewise benefit the community. Any efficacious antimalarial reduces the duration of infectiousness compared with an untreated or partially treated infection by killing asexual parasites, the source of gametocytes which are the transmissible life stage of the parasite. Drugs with gametocytocidal action further reduce the duration of infectiousness. Gametocytocidal drugs may be effective only during part of the parasite life cycle; for example, artemisinin derivatives act against immature gametocytes but are not effective against late-stage gametocytes^6^.

DHA–PQP has recently been introduced into national guidelines as an option for first- or second-line treatment in several African countries, including Ghana, Senegal, Kenya and Nigeria, and is in trials in several further countries. AL currently dominates the antimalarial market, comprising 77% of the 331 million ACT treatments used in 2012 (ref. 7). AS–AQ and AS–SP are also used for first-line treatment in some African countries but are not suitable for all areas due to resistance to the partner drugs. AS–MQ has not had wide uptake in Africa and is not included in the treatment policy of any sub-Saharan African country^7^. We therefore focus on contrasting DHA–PQP with AL as a first-line treatment option. The piperaquine component of DHA–PQP has a longer half-life than lumefantrine^8^, therefore DHA–PQP is likely to provide greater post-treatment prophylaxis, although the duration of protection is not known. A recent analysis based on reinfection rates after treatment with DHA–PQP or AL in moderate-to-high-transmission areas in children estimated that 12% of cases could be prevented by using DHA–PQP, and that using DHA–PQP as first-line treatment could save costs while averting more cases^9^. Here, we additionally consider gametocytocidal effects, treatment in adults, variations in transmission intensity and access to treatment across malaria-endemic countries in Africa. DHA–PQP has a weaker effect against gametocytes and infectivity to mosquitoes than AL^10^,^11^, which may be due to less frequent dosing and a lower total dose of the artemisinin component. In addition, lumefantrine may inhibit parasite development in the mosquito^12^,^13^. Here we develop and parameterize pharmacokinetic-pharmacodynamic (PKPD) models for DHA–PQP and AL, which are then embedded within a malaria transmission model. Second, we use the model to simulate the impact and cost effectiveness of replacing AL with DHA–PQP as first-line treatment in malaria-endemic countries in Africa, taking into account different transmission patterns, access to treatment and health system costs. We estimate that the difference in impact in terms of reducing clinical episodes between the two treatments would be modest in most malaria-endemic areas of Africa, but that DHA–PQP has a higher impact in the majority of the population at risk. Where transmission is very low, AL has the same or higher impact as DHA–PQP due to its greater gametocytocidal effects. Despite the small difference in the impact of the two drugs, the cost implications are substantial given that the estimated cost per case averted by using a locally appropriate treatment compares favourably with other malaria control interventions.

## Results

### Pharmacodynamics of piperaquine and lumefantrine

The duration and extent of protection provided by DHA–PQP and AL drug regimens has not been characterized in detail. To estimate this, we analysed data on reinfection after treatment of uncomplicated malaria with DHA–PQP or AL from randomized clinical trials^14^,^15^. We incorporated published pharmacokinetic models; piperaquine capillary blood concentrations were simulated^8^ based on data from children in Africa aged 2–10 years. We used a pharmacokinetic model of lumefantrine with a two-compartment structure^16^. The artemisinin components have very short half-lives of just a few hours so do not contribute to prophylaxis. Both lumefantrine and piperaquine act against blood-stage infection. We therefore modelled the protective effect of treatment as a reduced probability of new blood-stage infection becoming established in the presence of a given concentration of antimalarial. The concentration-effect curve takes the form:


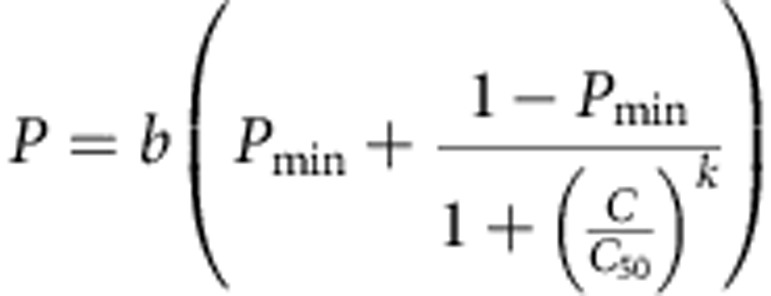


where *b* is the infection probability (the probability of an infectious bite leading to a slide-positive blood-stage infection when drug concentration is zero), *bP*_min_ is the infection probability at infinitely high blood drug concentration, *C* is the blood drug concentration, *C*_50_ is the blood drug concentration that gives half the maximum reduction in the infection probability and *k* determines the steepness of the concentration-effect curve.

We simulated reinfection after treatment as in a previous analysis^17^, incorporating age, heterogeneity in exposure to mosquito bites and pre-erythrocytic immunity. The model was fitted to the proportion of individuals reinfected over time in the clinical trials by varying the parameters of the concentration-effect curves, which were assumed to be the same across sites, and the entomological inoculation rate (EIR) in each site. We fitted the data from the different sites and trial groups simultaneously, using Bayesian Markov Chain Monte Carlo methods with a random walk Metropolis–Hastings algorithm to sample from the posterior distribution.

**Figure 1:**
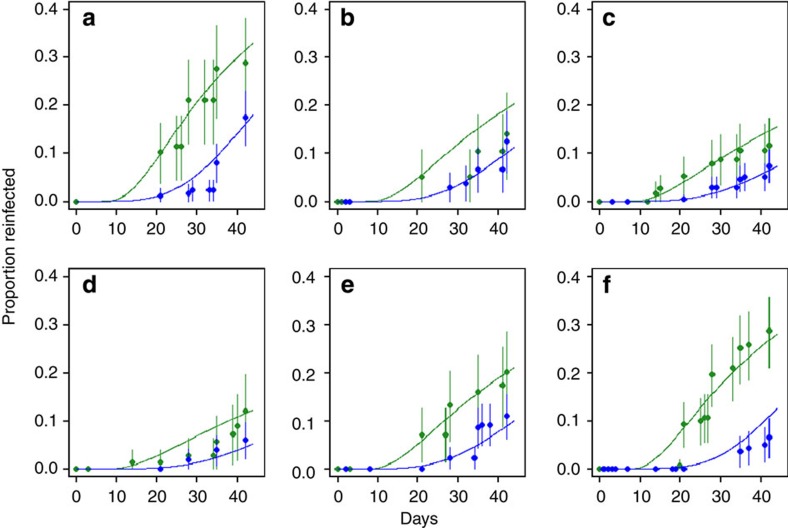
PKPD model fit. Model predictions (lines) and cumulative PCR-confirmed reinfection rates in clinical trial data in 1,651 individuals in six sites (points with 95% CI). Green=AL and blue=DHA–PQP. Sites: **a**=Nanoro, Burkina Faso; **b**=Kilifi, Kenya; **c**=Manhiça, Mozambique; **d**=Mbarara, Uganda; **e**=Ndola, Zambia^14^; and **f**=Bobo-Dioulasso, Burkina Faso^15^.

**Figure 2:**
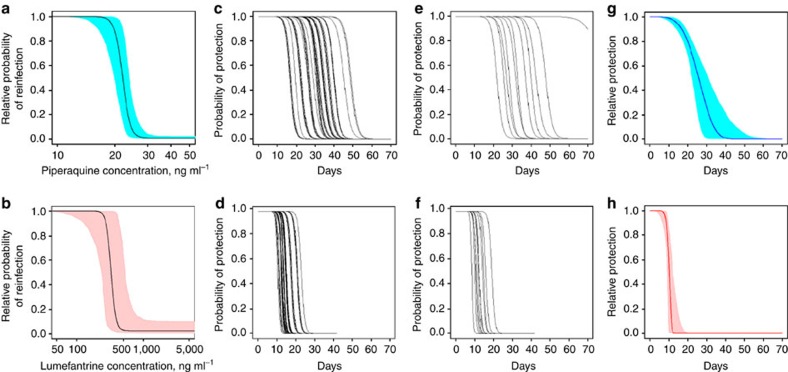
PKPD results. Concentration-effect curves for piperaquine (**a**) and lumefantrine (**b**) estimated from model fitting, with 95% CI. Piperaquine concentrations relate to capillary measurements and lumefantrine concentrations to venous measurements. Probability of protection from reinfection over time since the first dose based on pharmacokinetic models: piperaquine (**c**,**e**) and lumefantrine (**d**,**f**), simulations in children <10 years in the clinical trials (**c**,**d**) and in all age–weight groups based on Tanzanian bodyweight distribution (**e**,**f**). Probability of protection from reinfection over time since the first dose with piperaquine (**g**) or lumefantrine (**h**)—model fits and 95% CI using a Weibull survival function instead of pharmacokinetic models.

The cumulative rate of PCR-confirmed reinfection in the clinical trial data was generally higher in children treated with AL compared with DHA–PQP (Fig. 1), with the difference tending to be larger in higher transmission sites due to more reinfection events. The best-fitting model predictions from our PKPD model were within the 95% confidence intervals (CI) of the large majority of data points (Fig. 1). The concentration at which each antimalarial prevents 50% of new infections from successfully establishing as blood-stage infections was estimated at 22.1 ng ml^−1^ for piperaquine and 332.3 ng ml^−1^ for lumefantrine (Fig. 2a,b; Table 1). In the study populations in which the clinical trials were conducted, the mean number of days for which piperaquine prevented 90% or more reinfections was 26.2 (range 13.6–45.0 days depending on dose–weight group) and it prevented 50% or more reinfections for 29.4 days (range 16.4–48.8 days; Fig. 2c). Lumefantrine was estimated to provide over 90% protection for 12.1 days (range 9.0–20.6 days) and over 50% protection for 13.8 days (range 10.2–22.8 days; Fig. 2d). The duration of protection provided by both drugs varied by bodyweight, dose and age, but was particularly variable for piperaquine, in line with variable efficacy results by dose group^18^. The data on reinfection covered 42 days after treatment, while piperaquine was estimated to give some protection after 42 days in weight groups who have higher piperaquine exposures (Fig. 2c,e). However, in these age groups, the curves after 42 days are informed by dose–weight groups in whom piperaquine concentrations decline more quickly, reaching low levels within 42 days. We extended pharmacokinetic simulations of piperaquine and lumefantrine concentrations to all age groups using published age–weight relationships, and pharmacokinetic studies in adults, where available (Fig. 2e,f; Supplementary Methods; Supplementary Fig. 2).

Since there is uncertainty in the pharmacokinetic models, particularly in extrapolating to different populations and age groups, we also undertook a simpler analysis without pharmacokinetics in which the probability of protection against reinfection after treatment *P* was assumed to decline according to a Weibull survival curve (see Methods). This analysis estimated that the duration of protection at a level of 90% or higher was 16.8 days for piperaquine and 8.6 days for lumefantrine, and the duration of protection at 50% or over was 25.8 and 10.2 days, respectively (Fig. 2g,h). These are in line with the results of the PKPD model, although the piperaquine protection declines more gradually because it is an average of the weight-specific estimates (Fig. 2c,d). Supplementary Table 1 gives further details of the sensitivity analysis of the PKPD results.

AL and DHA–PQP have different effects on gametocytes and onward transmission after treatment^19^. We assumed that patients infected 1.85 times more mosquitoes when treated with DHA–PQP than with AL based on a human-to-mosquito transmission study^11^.

### Validation of transmission and treatment model

Using the relationships between drug concentrations and post-treatment prophylaxis estimated in the PKPD analysis, we simulated the potential impact of using AL or DHA–PQP as first-line treatment in endemic populations. We used an existing age-structured individual-based mathematical model, which describes the full transmission cycle of the parasite between humans and mosquitoes, as well as disease progression in humans, and has been detailed elsewhere^17^,^20^. The model has been fitted to extensive data on parasite prevalence via microscopy and PCR and episodes of uncomplicated malaria by age and transmission setting across Africa. It also incorporates the impact of long-lasting insecticide-treated nets (LLINs). We developed the model to include the estimated dose–weight-specific post-treatment prophylactic profiles (Fig. 2e–h).

We first validated our transmission model by comparing simulations with data from a long-term trial in Tororo, Uganda, in which children aged 4 months–1 year were randomized to receive AL or DHA–PQP every time they presented with malaria at health facilities and were followed up until age of 4 years^21^. The rate of clinical episodes in the DHA–PQP group in the trial was 0.84 (95% CI 0.74–0.95) times the rate in the AL group. The model-predicted rate ratio of clinical incidence, taking into account the local transmission intensity in the trial area and the seasonal variation, was 0.87, within the 95% CI of the trial estimate. The rate ratio was not substantially affected by assuming a high or low LLIN coverage.

### Influence of transmission intensity and seasonality on treatment impact

**Figure 3:**
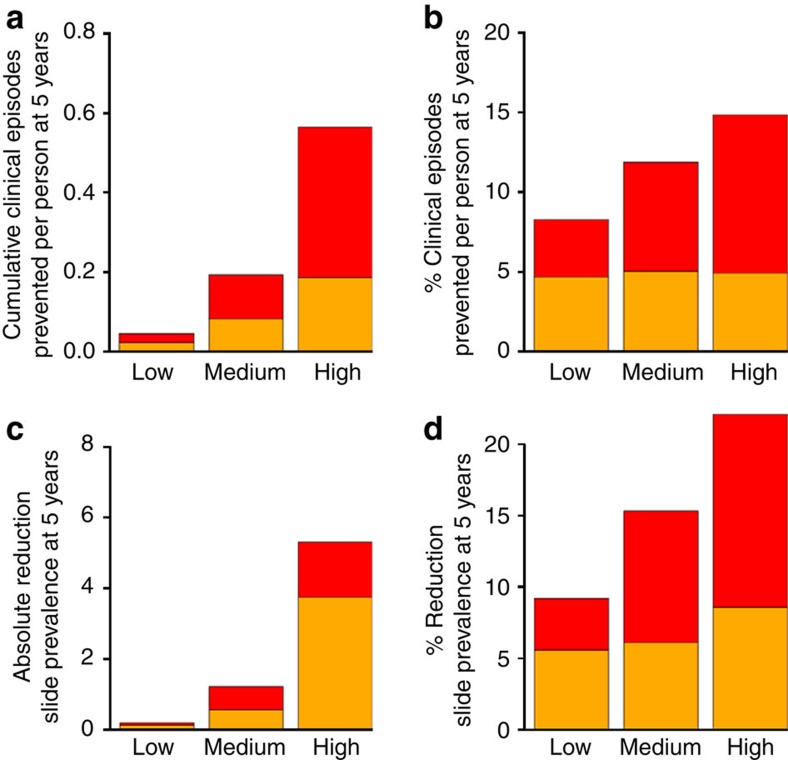
Generic model simulations. Model-simulated impact in all age groups on clinical episodes and parasite prevalence of having DHA–PQP as first-line treatment versus AL over 5 years in low, medium and high transmission settings with (red) and without (orange) seasonal variation in transmission, assuming high treatment access (80% of cases are treated), but no other interventions. Low, medium and high indicate baseline slide prevalence levels before treatment change of 5, 15 and 50%, respectively, in children aged 2–10 years in the non-seasonal setting. Seasonal settings have the same baseline clinical incidence as the non-seasonal settings. Absolute reductions (**a**,**c**) and percentage reductions (**b**,**d**) in the DHA–PQP versus AL scenarios are shown.

Generalized simulations of switching treatment policy from AL to DHA–PQP were run to explore the impact in areas with different initial transmission intensity (50, 15 and 5% baseline slide prevalence of malaria in 2–10 year olds) and seasonality in transmission (uniform over the year or strongly seasonal with ~90% of infectious bites occurring within 4 months). Here we assumed that 80% of clinical cases would be treated with AL, switching all treatment to DHA–PQP at a given time point. Outcomes of cumulative number of clinical episodes prevented and reductions in slide prevalence were assessed 5 years after the change in treatment policy in all age groups, comparing this with a continuation of the AL policy. In the scenarios considered, DHA–PQP reduced transmission compared with AL, indicating that its longer post-treatment prophylactic period was more important than the higher gametocytocidal effect of AL (Fig. 3). Reductions in transmission were higher in areas with high initial transmission intensity due to the greater chance of receiving an infectious bite during periods of post-treatment prophylaxis. For example, in non-seasonal settings, an estimated 0.03 and 0.19 clinical episodes were prevented per person when initial slide prevalence was 5 and 50%, respectively (Fig. 3a). Approximately 82 and 26% of this impact is due to the direct protection by the drug in the highest and lowest transmission settings, respectively, the remainder being due to a community-wide effect on transmission. Estimated impacts were always higher in areas with seasonal variation in transmission. For example, an estimated 0.19 episodes were prevented per person in a non-seasonal setting with baseline slide prevalence of 50% versus 0.56 episodes in a seasonal setting with the same total annual clinical incidence.

### Estimated impact of treatment across Africa

Transmission model simulations were run at the resolution of the first administrative unit across Africa (Supplementary Fig. 3), using three types of specific local data for each area: (1) the underlying population demographic data^22^,^23^ combined with the slide prevalence in 2010 (ref. 24) (Supplementary Fig. 3a), (2) the seasonal pattern determined by high-resolution rainfall data^25^ (Supplementary Fig. 3b) and (3) intervention coverage, that is, current access to treatment and LLIN coverage (Supplementary Fig. 3c,d)^7^,^26^,^27^. We used a published analysis of treatment access by administrative unit to obtain the estimated proportion of fevers treated with an antimalarial, the proportion of these antimalarials that are ACTs, and whether treatment is sourced in the public versus private sector^27^. In each administrative unit, we simulated transmission from 2000 to 2017, matching 2010 prevalence to the most recent Malaria Atlas Project map^24^. We incorporated location-specific data on LLIN scale-up up to 2012 and assumed that coverage remained at the 2012 level in future years. We simulated the introduction of either AL or DHA–PQP as first-line treatment at the beginning of 2012 and calculated the cumulative clinical incidence over the next 5 years. In each case, we assume that all ACTs taken are either AL or DHA–PQP. We simulated various different scenarios about coverage of AL and DHA–PQP in each area.

**Figure 4:**
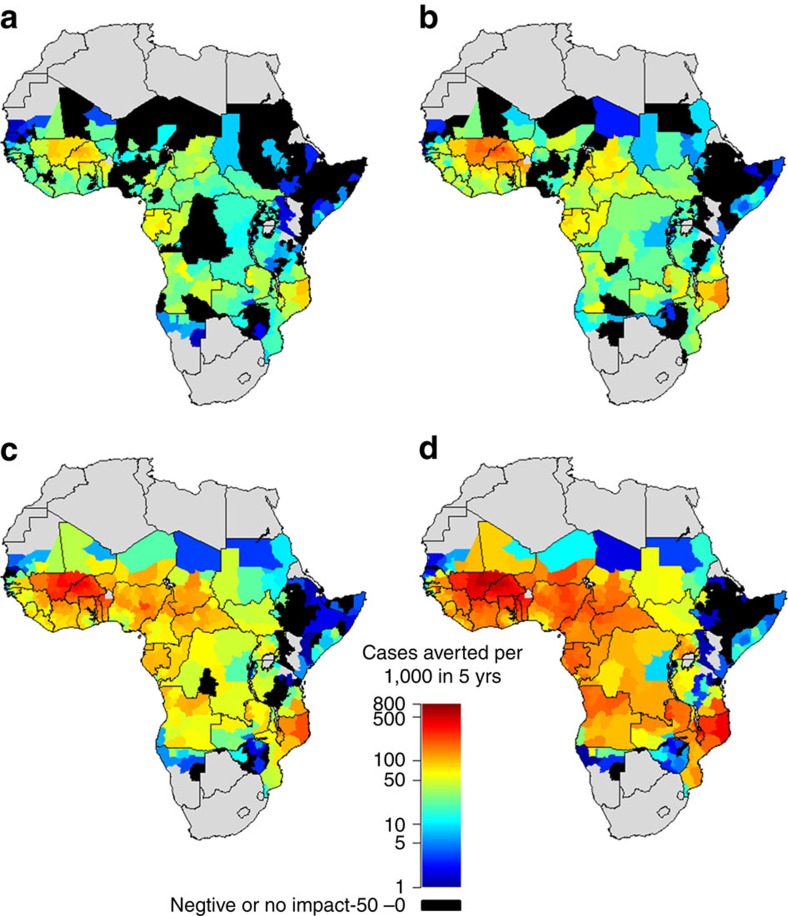
Africa-wide simulations. Estimated impact of using DHA–PQP versus AL as the first-line treatment by first administrative unit in malaria-endemic areas of Africa. Cumulative numbers of clinical episodes prevented 5 years after changing treatment policy per 1,000 individuals of all ages, under different coverage scenarios: (**a**) current ACT treatment rates in the public sector only, (**b**) current ACT treatment rates in the public and private sector, (**c**) current antimalarial treatment rates with scaled-up ACT coverage to 100% and (**d**) scaled-up treatment access—80% of clinical malaria cases receive ACT. Grey areas indicate no *P. falciparum* or a slide prevalence <1% or no data.

Assuming that the proportion of cases receiving an antimalarial and ACT coverage remains at their current levels^27^ (Fig. 4a) and that treatment policy change would only affect the public sector, DHA–PQP had a modestly higher impact than AL in 64% of the population at risk. In these populations, a median of 26 cases per 1,000 people (interquartile range (IQR): 15–43) were averted in the 5 years after the policy change compared with the scenario of using AL. Areas where DHA–PQP was predicted to have the highest impact were those with high levels of transmission, large seasonal variation in transmission and with reasonable ACT coverage in the public sector; these included Burkina Faso, southern Mali and northeast Mozambique, where the number of cases averted was up to 100 per 1,000 people over 5 years. In a further 32% of the population at risk, there was negligible difference (<0.5%) between the AL and DHA–PQP scenarios. In some areas, this was simply due to low recorded ACT coverage. In other areas such as the highlands of East Africa, there was less impact due to low malaria transmission, meaning that there is less benefit of prophylaxis. In 4% of the population at risk, AL had a slightly better impact than DHA–PQP; these were areas with low endemicity, where there is almost no benefit of prophylaxis, but there is some benefit of the greater gametocytocidal action of AL. The difference was small, with a median of 2.7 (IQR: 1.8–6.3) fewer cases per 1,000 over 5 years in the AL compared with DHA–PQP scenario. We also ran a subgroup analysis for countries that currently use AL as a first-line treatment^28^. An estimated 7.3 million clinical cases would be averted over 5 years by switching to DHA–PQP, or 0.9% of total cases (IQR across areas: 0.4–1.7%). This assumes that all ACTs used were either AL or DHA–PQP.

Simulations, which assumed that a change in treatment policy would cause a change in the ACTs used in the private as well as public sector (Fig. 4b), showed, as would be expected, a larger difference in impact between the two ACTs, particularly in countries with significant use of ACTs in the private sector, such as Burkina Faso, Rwanda and Burundi. In countries with an AL first-line policy, the total estimated number of cases averted by using DHA–PQP instead of AL over 5 years was 11.9 million, 1.4% of all cases. Impact on EIR and slide prevalence are shown in Supplementary Figs 4 and 5. Due to uncertainty in estimates of ACT coverage, we ran the analysis of policy change in public and private sectors twice using two different sources of data: an analysis by Cohen *et al*.^27^ and the World Malaria Report^28^. For those areas where ACT coverage data were available from both sources, the difference between DHA–PQP and AL was larger using the World Malaria Report data due to higher net ACT coverage estimates relative to local malaria case numbers, with an estimated 2.2% of all cases averted by using DHA–PQP instead of AL, as opposed to 1.4% of cases using the Cohen *et al*. estimates.

If ACT coverage were scaled up to 100% in both public and private sectors, and the proportion of cases receiving an antimalarial remained at current levels, the estimated difference in impact between DHA–PQP and AL in countries currently using AL increased to 33.5 million cases averted, 4.0% of the total (Fig. 4c). The higher impact was marked in areas with low reported current ACT coverage, for example, in Nigeria. If both treatment access and ACT coverage were scaled up so that 80% of clinical malaria cases received an antimalarial with 100% ACT coverage, the impact of DHA–PQP increased to an estimated 57.6 million cases averted over 5 years, 7.4% of the total (Fig. 4d; IQR across areas: 4.7–8.3%). Under these assumptions, the proportion of the population at risk who experienced a positive impact of DHA–PQP increased to 93%. The maximum impact range under this scenario was 10–15% of clinical cases averted in high-transmission areas with high levels of seasonal variation, such as Burkina Faso. Eight percent of the population at risk had estimated case reductions of >10%. With equal treatment access across areas, the strongest predictor of whether DHA–PQP would have a greater impact than AL was transmission intensity. Sixty-four percent of areas where AL had equal or better impact than DHA–PQP had a slide prevalence of <5% in 2–10 year olds, whereas 92% of areas with a positive impact of DHA–PQP had a slide prevalence of >5%.

### Cost effectiveness

**Figure 5:**
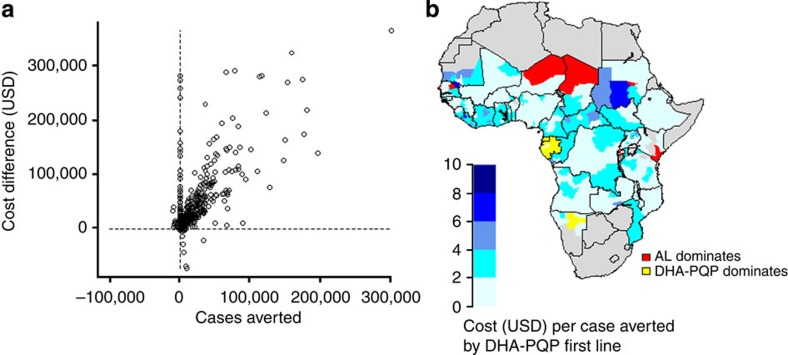
Cost effectiveness. (**a**) Total cases averted and total difference in costs of treatment in USD over 5 years in 492 administrative areas of Africa, comparing use of DHA–PQP with AL. (**b**) Incremental cost-effectiveness ratios: cost per case averted by introducing DHA–PQP as the first-line treatment instead of AL, averaged over 5 years by first administrative unit. Blue scale=areas where DHA–PQP has a positive impact on averting cases but higher overall cost than AL, red=AL dominates (averts more cases with lower costs than DHA–PQP), yellow=DHA–PQP dominates. Grey areas indicate no *P. falciparum* or *P. falciparum* slide prevalence <1% or no data.

We calculated the incremental cost-effectiveness ratio in each area over a 5-year period based on the predicted cumulative number of clinical malaria cases in all age groups with DHA–PQP versus AL as first-line policy. Our main cost-effectiveness analysis focusses on the scenario with current treatment access and ACT coverage in the public sector (Fig. 4a). In 64% of the population at risk, DHA–PQP improved impact on transmission but there was a higher cost of treatment due to slightly higher unit costs of DHA–PQP compared with AL (Fig. 5; Table 2). In these areas, the median incremental cost per case averted over 5 years after changing treatment policy was $1.94 (IQR: $1.20–2.75). In areas with negligible difference between the clinical incidence under the DHA–PQP scenario and the AL scenario, the cost was on average 7% less in the scenario where AL was used. In 0.4% of the population at risk, DHA–PQP reduced both the total number of cases and total costs. This occurred where the case reduction was sufficiently high and in countries where the cost of an outpatient visit is relatively high (>$7 per appointment), so that antimalarial costs constituted only a small part of the total cost of treating a malaria case. These were areas in Gabon, Equatorial Guinea and Namibia. The cost was on average 0.7% less than that in the scenario where AL was used. Where clinical incidence was higher under the DHA–PQP scenario, a DHA–PQP policy was not incrementally cost effective. In these areas, the median difference in case numbers between the AL and DHA–PQP scenarios was 1.0%, while the cost was 11% cheaper with AL than DP. In the scenario with scaled-up access to treatment (80% of cases receive an ACT), areas with >10% case reductions had an estimated cost reduction of 4% under the DHA–PQP scenario versus the AL scenario due to fewer outpatient visits.

### Drug choice versus treatment access and coverage

We also estimated the impact of increasing ACT coverage and access to treatment on total clinical burden to determine the importance of these factors relative to the drug choice between DHA–PQP versus AL. In countries which currently use AL as a first-line treatment, increasing AL coverage to 100% among patients who receive an antimalarial, while assuming no change in the proportion of cases receiving an antimalarial, predicts that 2.5 million (0.3%) cases would be averted compared with the scenario of keeping ACT coverage at current levels. This impact is therefore lower than switching to DHA–PQP and keeping current ACT coverage the same (see above: 7.3 million cases averted (0.9%)). However, if the proportion of cases that receive an antimalarial increased to 80% in these countries and all patients received AL, an estimated 59.8 million cases would be averted (7.2% of total; compared with 117.4 million total averted under a DHA–PQP policy).

## Discussion

Using two common first-line treatments for malaria in Africa—AL and DHA–PQP—as examples, our analysis demonstrates the complexity of assessing the potential impact and cost effectiveness of new treatments in settings that vary in malaria epidemiology, access to care and associated healthcare costs. While our analysis was not intended to recommend a particular antimalarial, for which other factors including the potential side effects, risk of resistance and patient adherence are also important, it does illustrate the potential advantages and disadvantages of key drug properties including their gametocytocidal efficacy and duration of post-treatment prophylaxis relative to the drug cost.

Overall, we predict a modest average reduction in clinical malaria incidence in almost two-thirds of the simulated population at risk in endemic African settings under current estimates of ACT access if first-line treatment is switched to DHA–PQP due to its longer post-treatment prophylaxis. The benefit was greater in high-transmission areas. Our estimates of 10–15% reduction in cases in such areas when treatment access is high are in line with the Pfeil *et al*.^9^ estimate of 12% of cases averted by using DHA–PQP as first-line treatment rather than AL in children in moderate-to-high-transmission areas. However, in the majority of endemic areas, our estimated impact of DHA–PQP is lower than that of Pfeil *et al*. due to considering lower-transmission areas, lower treatment access and treatment in adults who have greater immunity. A similar impact of post-treatment prophylaxis would be predicted with other long-acting partner drugs, such as mefloquine. However, in about a third of the population at risk living in lower-endemicity areas, the advantage of a longer prophylactic period after DHA–PQP was less important. Because AL has a larger impact on transmissibility, AL either produced a better or similar reduction in clinical incidence.

Although the difference in impact between the drug regimens was generally small, there were important cost implications given the current lower price of AL compared with DHA–PQP. The incremental cost per case averted in areas where there was a benefit of DHA–PQP was low given that a new first-line treatment would be provided through existing primary-care facilities, with a median of $1.94. This compares with a median cost per case averted of other malaria control interventions (under a provider perspective) of $24 (range $2.29–71) for insecticide-treated nets, $19 (range $0.54–267) for indoor residual spraying^29^, $0.68–2.27 for intermittent preventive treatment of infants with SP^30^ and $64.93–208.69 for seasonal intermittent preventive treatment^31^. However, we did not factor in the cost of changing treatment policy as this is difficult to quantify and would vary across countries. As in the previous Pfeil *et al*. cost-effectiveness analysis, DHA–PQP could in some areas reduce both the total number of cases and total costs; however, our analysis found that this applies only in a very small number of areas (0.4% of the population at risk). In areas where AL has a similar or better impact, using this less-expensive drug saves on treatment costs. Antimalarial prices fluctuate and we did not take into account of this variation. We did not include the cost of co-administering AL with milk or other fatty substance, which is required to aid absorption, and may be provided by public health facilities at ~$0.48 per treatment course^32^.

Perhaps, surprisingly, the better gametocytocidal effect of AL did not produce an improved impact in our simulations in many areas when compared with a drug with a longer half-life. Both regimens are highly efficacious in rapidly clearing parasites from the blood and hence at reducing transmission. Therefore, although the infectivity may be around 2-fold higher after DHA–PQP, both AL and DHA–PQP reduce the average duration of infectiousness more than 50-fold compared with untreated infections (Table 1). Given that only a proportion of malaria infections in the population are treated, the majority of transmission arises from untreated infections, and therefore this small difference in infectiousness among treated individuals rarely has a measurable impact in our model. This result is dependent on our assumptions about the relative infectiousness of treated and untreated cases. Our parameters underlying this are derived from a combination of studies^11^,^33^,^34^,^35^. Sensitivity analysis indicates that assuming a higher infectivity of treated individuals compared with untreated individuals (but the same relative infectiousness of DHA–PQP patients versus AL patients) would slightly increase the range of settings in which AL has a better impact than DHA–PQP (Supplementary Fig. 6).

Several countries (Ghana, Kenya, Nigeria and Senegal) are introducing multiple first-line therapies for malaria to help combat drug resistance. Given that there are five recommended ACTs, there is still scope to choose regimens that suit the local epidemiology as long as partner drug resistance does not limit the options. For example, AS–SP, AS–MQ and DHA–PQP are all longer-acting regimens^36^. The spatial scale over which multiple first-line therapies should be implemented remains unclear; however, if implemented regionally, longer-acting ACTs could be targeted to areas of higher transmission, while lower-transmission areas could receive ACTs optimal in terms of cost or gametocytocidal effects. This would be consistent with wider policy moves away from a ‘one size fits all’ approach to malaria control.

While our analysis finds that choosing a locally appropriate treatment is important, impact is limited by current low access to treatment in some areas. In countries which currently have an AL first-line policy, we estimate that increasing access so that 80% of cases received AL would avert over sevenfold more cases than switching policy from AL to DHA–PQP with no change in current coverage levels. Likewise, the impact of an appropriate treatment is dramatically increased when coverage is high.

Resistance and patient adherence are additional important considerations when selecting treatment policy, which we did not include in our analysis. Piperaquine resistance is not reported in Africa, but has been reported in Asia^37^,^38^,^39^. The long half-life of the drug may make resistant parasites spread more quickly than any lumefantrine-resistant parasites^40^. Clinical resistance to lumefantrine has not been officially confirmed, although there is likely to be cross-resistance with mefloquine^41^,^42^,^43^. Patient adherence to the treatment regimen is likely to be better with fewer doses and a simpler regimen: on this metric, the three-dose DHA–PQP regimen has an advantage over the six-dose AL regimen^44^. Furthermore, the need for sufficient fat intake to optimize AL absorption may lead to reduced efficacy and a shorter prophylactic period for lumefantrine^45^.

Our analysis made a number of simplifying assumptions; in particular we assumed that treatment was only taken by symptomatic malaria cases. In reality, lack of laboratory diagnosis means that antimalarials are often taken by individuals with either asymptomatic or no parasitaemia. This treatment also has an impact on transmission through reducing transmission from asymptomatic cases and providing prophylaxis. Sensitivity analysis indicates that we may therefore underestimate the difference in impact of the two drugs (Supplementary Fig. 7). However, with improved use of and adherence to the results from rapid diagnostic tests, uninfected individuals may be less likely to receive treatment in the future. In several countries, ACTs are only given to confirmed cases. Treatment among uninfected individuals is likely to vary by endemicity and age as well as treatment access. Further work is needed to define these patterns. Second, we did not include the possibility that long-acting drugs could suppress parasite density in new infections without completely clearing them, so that individuals may still transmit if they have a subpatent infection acquired during the elimination period of the drug. We were not able to assess this in the absence of more sensitive molecular detection of reinfection. Third, while we used the most recent available survey data, for some countries this is out of date.

Increasing access to ACTs remains the most important overall goal with regards to treatment for malaria control. However, with five different ACTs to choose from, our analysis suggests that choosing an appropriate treatment for the local area often results in a more incrementally cost-effective intervention than vector control. The longest-acting regimens have high impact in areas with higher transmission, particularly when there is seasonal variation. In areas of lower transmission (baseline slide prevalence <5% in 2–10 year olds), we find that there is less advantage of investing in a long-acting drug regimen and here effective, cheaper drug regimens could be prioritized. More generally, our analysis suggests that there is only a small additional benefit to increasing the gametocytocidal action of a first-line treatment above the level achieved by current ACTs, when most transmission arises from untreated cases. Considering the relative benefits of different drug actions could aid policy makers and drug developers in prioritizing investment.

## Methods

### Ethics statement

All data analysis conducted during this research was secondary and used studies that had obtained ethical approval previously from the appropriate organizations. All data were anonymized before being provided to investigators.

### Data

Data were from two randomized clinical trials of DHA–PQP and AL in six different African sites, whose participants were children >0.5 years with *P. falciparum* mono-infection (1,651 individuals with 14,241 observations)^14^,^15^. Individuals were tested for parasitaemia up to day 42 after treatment. Insecticide-treated nets were given to patients in some sites, which was taken into account in our analysis (see below). We excluded individuals with recrudescent parasitaemia, since reinfection status could not be assessed. We also excluded those who did not follow the trial protocol and those >10 years old (see Supplementary Methods for details of inclusion criteria).

### PKPD models

We categorized individuals into groups according to site, bodyweight (starting at 5 kg, individuals are grouped by 2 kg increments up to 27 kg, which in turn determines dose) and exposure. We simulated the pharmacokinetics of piperaquine and lumefantrine in each of these groups using the mean bodyweight and dose in the group and existing models^8^,^16^. When extending simulations to adults, we used a published pharmacokinetic model of venous piperaquine concentrations^46^ to estimate capillary concentrations in adults^8^. This provided very high estimates of protection for piperaquine in adults (>42 days of >50% protection; Fig. 2e). Since the relationship between venous and capillary concentrations is variable, and the PK model for adults was based on a small PK study in women in Thailand, it was uncertain how representative these would be of an adult population in Africa. Therefore, we set the adult post-treatment profile to equal that of the non-PK-based analysis in children (Fig. 2g) and the outlying profile in Fig. 2e was not used. Lumefantrine pharmacokinetics are ideally described using a model with at least two compartments^16^. There is not yet such a model fitted to pharmacokinetic data in children in Africa. We therefore used a pharmacokinetic model of lumefantrine based on data in pregnant women in Asia^16^ and adapted the model parameters to match a published pharmacokinetic data set in African children^47^ as follows. We started with the original parameter values, then assumed the following parameters to be directly proportional to bodyweight: the central volume of distribution, the clearance rate from the central compartment, the intercompartmental clearance rate and the peripheral volume of distribution. We simulated the three dose groups in the pharmacokinetic study^47^ using their mean bodyweights. The predicted venous plasma concentration across dose groups was fitted to the data using least squares, by further scaling the two clearance parameters by a single allometric parameter, which was estimated (Supplementary Fig. 1). We included interindividual variation in the pharmacokinetic parameters and compared this with the population average results. For the piperaquine simulations, we used the random effects fitted in the original model and for the lumefantrine simulations, we assumed the same percent coefficient of variation (CV) on each parameter as in the original fit of the model to the data from adults (where 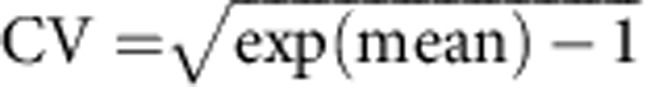). We numerically integrated over the random effects using multivariate Gauss–Hermite quadrature with four quadrature points per parameter.

We also estimated protection against reinfection *P* after treatment as a Weibull survival curve without any pharmacokinetic assumptions, assuming no difference by age or weight:


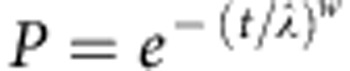


where *t* is time, *λ* is a scale parameter and *w* controls the slope of the curve. *λ* and *w* were estimated for each antimalarial regimen.

We simulated reinfection after treatment as described^17^ in each dose–weight group according to the local age structure^22^, assuming a constant force of infection over time. We also explored using a seasonally varying EIR specific to each of the study sites, based on rainfall pattern as done previously^25^. Exposure to bites was assumed to be reduced by 50% for those sites where participants were given an insecticide-treated net at recruitment into the trial^48^,^49^. If parasites emerging from the liver survive, we assumed a 3.5-day time lag^50^ until they become detectable by microscopy. During model fitting, all available individual observations were used to calculate the Binomial log likelihood of the model given the probability of reinfection during a time interval since the individual was last tested, for each dose–weight group.

We used log-normal prior distributions for the annual EIRs in each site based on values in the literature where available (Table 1). For three sites, EIR data were not available, so the EIR was estimated from slide prevalence based on a published relationship^17^ and the prior distribution was assigned a larger s.d. to allow for greater uncertainty (Table 1). Without a reasonably informative prior, the EIR is difficult to distinguish from the concentration-effect curve.

Half-normal prior distributions with large variances were used for the pharmacodynamic parameters *C*_50_ and *k* (Table 1). The *C*_50_ prior distributions were informed by clinical trials, which found a cut-off on the day 7 concentrations at which subsequent reinfection was more likely, but were only weakly informative to cover the plausible biological range^8^,^51^. The infection probability at maximum drug concentration was constrained to be small for both piperaquine and lumefantrine, since there is currently no significant documented failure of either of these drugs in Africa^19^ (Table 1).

### Model simulations

We used an existing age-structured individual-based mathematical model^17^,^20^ and modified this to include PKPD. We validated our model against a trial in Tororo, Uganda, comparing AL with DHA–PQP^52^. In simulations of this setting, we matched the slide prevalence according to Malaria Atlas Project 2010 estimates and the treatment intake as observed in the trial (6.4 treatments per person-year in the AL arm and 5.3 in the DHA–PQP arm). We simulated clinical incidence in the 0.5- to 4-year old age group over the 3.5 years of the intervention. Simulated coverage with LLINs in the community was varied.

In the Africa-wide simulations, the proportion of symptomatic malaria infections that are treated with effective antimalarials is an important parameter for determining treatment impact. Data from Demographic and Health Surveys^53^ on the proportion of fevers that are treated with a specific type of antimalarial in the public versus private sector has recently been aggregated and analysed from each country and administrative unit^27^. Direct data were available for children under 5 years of age for 421 administrative units and were standardized for the month of survey. A relationship between the rate of treatment in children under 5 and those aged over 5 was determined by the authors from 61 surveys of treatment rates in all ages. We assumed that the probability of treatment of fever cases with antimalarials was a good estimate of the probability of a symptomatic malaria case getting an antimalarial. We modelled only treatment taken by symptomatic malaria cases (not presumptive treatment taken by uninfected individuals). Symptomatic case incidence by age, time point and location is simulated by the existing transmission model.

We simulated the following scenarios about coverage of AL and DHA–PQP in each area:

1. The proportion of cases receiving an antimalarial and ACT coverage remains at their current level and we assume that only public sector treatment is switched from AL to DHA–PQP.
2. The proportion of cases receiving an antimalarial and ACT coverage remains at their current level and we assume that a change in national treatment policy would cause a change in the public and private sectors.
3. Scaled-up ACT coverage: the proportion of cases receiving an antimalarial remains at current levels but ACT coverage increases to 100% in public and private sectors and a change in national treatment policy operates in both sectors.
4. Scaled-up treatment access and ACT coverage: 80% of clinical malaria cases receive an antimalarial, with 100% ACT coverage (through either the public or private sector), and the switch to DHA–PQP occurs in both public and private sectors.

We excluded areas where the prevalence was under 1% from our analysis because the probability of local elimination of transmission in our stochastic model can skew the results. We used a population size of 10,000 as standard, and 100,000 in areas where slide prevalence was <10%, to smooth out stochasticity.

### Cost effectiveness

We calculated the unit cost per uncomplicated malaria case treated as the sum of the cost of the antimalarial^54^, the country-specific unit cost of an outpatient health facility visit^55^ and the cost of a rapid diagnostic test (Table 2). The unit cost of the antimalarial varies by dose and was calculated according to the number of cases occurring in each dose group, based on age-specific clinical incidence from the model and population age structure. All costs and health benefits were discounted annually at a rate of 3%^56^.

## Author contributions

P.H., D.U., L.C.O., M.B. and A.C.G. designed the study, L.C.O. wrote the first draft of the manuscript and L.C.O. and J.T.G. analysed the data with contribution from M.C. and J.T. All authors edited and commented on the manuscript.

## Additional information

**How to cite this article:** Okell, L. C. *et al*. Contrasting benefits of different artemisinin combination therapies as first-line malaria treatments using model-based cost-effectiveness analysis. *Nat. Commun.* 5:5606 doi: 10.1038/ncomms6606 (2014).

## Supplementary Material

Supplementary Information Supplementary Figures 1-7, Supplementary Tables 1, Supplementary Methods and Supplementary References

## Figures and Tables

**Table 1:** PKPD and reinfection model parameters.

**Parameters**	**Symbol**	**Prior distribution**	**Prior median (95% interval)**	**Posterior estimate and 95% credible interval, or fixed value**	**Units**	**Source**
*Fitted parameters, full PKPD model*						
Probability of reinfection as piperaquine concentration tends towards infinity	*P*_min_	Beta *α*=1, *β*=15	0.0045 (0.0018, 0.2184)	0.0038 (0.0001–0.0210)	—	19
Piperaquine capillary concentration that gives half the maximum reduction in the probability of blood-stage infection	*C*_50_	Half-normal, mean=0, s.d.=30 (absolute values)	20.2 (0.9, 67.2)	22.1 (19.9–24.1)	ng ml^−1^	8
Piperaquine power parameter	*k*	Half-normal, mean=0, s.d.=15 (absolute values)	10.1 (0.5, 33.6)	21.0 (9.5–40.7)	—	—
Probability of reinfection as lumefantrine concentration tends towards infinity	*P*_min_	Beta *α*=1, *β*=15	0.0045 (0.0018, 0.2184)	0.0224 (0.0006–0.1017)	—	19
Lumefantrine venous concentration at which gives half the maximum reduction in the probability of blood-stage infection	*C*_50_	Half-normal, mean=0, s.d.=500 (absolute values)	337.3 (15.6, 1121.2)	331.0 (229.2–543.4)	ng ml^−1^	51
Lumefantrine power parameter	*k*	Half-normal, mean=0, s.d.=15 (absolute values)	10.1 (0.5, 33.6)	12.2 (2.6–34.3)	—	—
Annual EIR—Nanoro, Burkina Faso	*ε*	Log-normal, mean=log* (130), s.d.=0.7	130 (33, 513)	97.3 (66.2–145.6)	ibpppy	14
Annual EIR—Kilifi, Kenya	*ε*	Log-normal, mean=log(34), s.d.=0.7	34 (9, 135)	19.6 (11.7–31.6)	ibpppy	14
Annual EIR—Manhiça, Mozambique	*ε*	Log-normal, mean=log(38), s.d.=0.7	38 (10, 150)	24.9 (16–39.3)	ibpppy	14
Annual EIR—Mbarara, Uganda	*ε*	Log-normal, mean=log(14.5), s.d.=0.8	14.5 (3, 70)	19.8 (11.5–34.3)	ibpppy	57,58
Annual EIR—Ndola, Zambia	*ε*	Log-normal, mean=log(6), s.d.=0.8	6 (1, 29)	41.1 (24.7–64.7)	ibpppy	58,59
Annual EIR—Bobo-Dioulasso, Burkina Faso	*ε*	Log-normal, mean=log(117), s.d.=0.8	117 (24, 561)	25.2 (17.1–36.5)	ibpppy	58
*Fitted parameters: model of prophylaxis without PKPD (Weibull survival curve)*						
Piperaquine scale parameter	*λ*	Half-normal, mean=0, s.d.=3,000 (absolute values)	84 (3.9, 280.2)	28.1 (23.6, 34.5)	Days	—
Piperaquine slope parameter	*w*	Half-normal, mean=0, s.d.=15 (absolute values)	10.1 (0.5, 33.6)	4.4 (2.9, 7.6)	—	—
Lumefantrine scale parameter	*λ*	Half-normal, mean=0, s.d.=3,000 (absolute values)	84 (3.9, 280.2)	10.6 (9.3, 13.1)	Days	—
Lumefantrine slope parameter	*w*	Half-normal, mean=0, s.d.=15 (absolute values)	10.1 (0.5, 33.6)	11.3 (4.0, 32.2)	—	—
Annual EIR—Nanoro, Burkina Faso	*ε*	Log-normal, mean=log*(130), s.d.=0.7	130 (33, 513)	74.4 (48.7–111.5)	ibpppy	14
Annual EIR—Kilifi, Kenya	*ε*	Log-normal, mean=log(34), s.d.=0.7	34 (9, 135)	17.9 (10.9–28.7)	ibpppy	14
Annual EIR—Manhiça, Mozambique	*ε*	Log-normal, mean=log(38), s.d.=0.7	38 (10, 150)	21.9 (14.5–33.2)	ibpppy	14
Annual EIR—Mbarara, Uganda	*ε*	Log-normal, mean=log(14.5), s.d.=0.8	14.5 (3, 70)	16.5 (9.6–27.6)	ibpppy	57,58
Annual EIR—Ndola, Zambia	*ε*	Log-normal, mean=log(6), s.d.=0.8	6 (1, 29)	32.7 (20.6–51.9)	ibpppy	58,59
Annual EIR—Bobo-Dioulasso, Burkina Faso	*ε*	Log-normal, mean=log(117), s.d.=0.8	117 (24, 561)	20 (13.6-28.9)	ibpppy	58
*Fixed parameters*						
Age-related biting parameter	*ρ*	Fixed	—	0.85		17
Age-related biting parameter	*a*_0_	Fixed	—	2920	Days	17
Variance in exposure to mosquito bites		Fixed	—	1.768	—	17
Infectiousness after AL treatment relative to an untreated infection		Fixed	—	0.05094	—	11,60
Infectiousness after DHA–PQP treatment relative to an untreated infection		Fixed	—	0.09434	—	11,60
Duration of treated infection		Fixed	—	5	Days	17
Duration of untreated infection		Fixed	—	195 patent infection followed by 84 subpatent infection	Days	17
*Pre-erythrocytic immunity*						
Time during which immunity cannot be boosted after a previous boost	*u*_b_	Fixed	—	4.44	—	17
Decay parameter	*d*_b_	Fixed	—	3650	Days	17
Infection probability in non-immunes	*b*_h_	Fixed	—	0.637	—	17
Lowest infection probability at maximum immunity relative to non-immunes	*b*_min_	Fixed	—	0.500	—	17
Scale parameter	*I*_B0_	Fixed	—	57.89	—	17
Shape parameter	*q*	Fixed	—	2.11	—	17

ibpppy, infectious bites per person per year; PKPD, pharmacokinetic-pharmacodynamic.

^*^Natural log.

**Table 2:** Cost input data.

**Item**	**Patient weight (kg)**	Patient age (years)*	**Cost in US$**	**Reference**
AL (one full treatment course)	5–14	0–4	0.42	Novartis price to GF AMFm†
	15–24	5–10	0.84	
	25–34	11–14	1.25	
	35+	15+	1.52	
DHA–PQP (one full treatment course)	5–13	0–3	0.67	Sigma-Tau price to GF AMfM‡
	13–24	4–9	0.93	
	24–36	10–14	1.46	
	36–75	15+	1.96	
RDT (one unit)	—	—	1.5	61

AL, artemether–lumefantrine; DHA–PQP, dihydroartemisinin–piperaquine; GF AMFm, Global Fund Affordable Medicines Facility-malaria; RDT, rapid diagnostic test.

^*^Range based on average age–weight relationship in endemic areas (see Methods).

^†^Niger public health sector, November 2012.

^‡^Cambodia public health sector, April 2012.
