# Supplementary material for: Contrasting benefits of different artemisinin combination therapies as first-line malaria treatments using model-based cost-effectiveness analysis
Source: Nat Commun. 2014 Nov 26;5:5606. doi: 10.1038/ncomms6606 (PMC4263185; doi:10.1038/ncomms6606)
Supplement: Supplementary Information — Supplementary Figures 1-7, Supplementary Tables 1, Supplementary Methods and Supplementary References [file ncomms6606-s1.pdf]

Supplementary figures

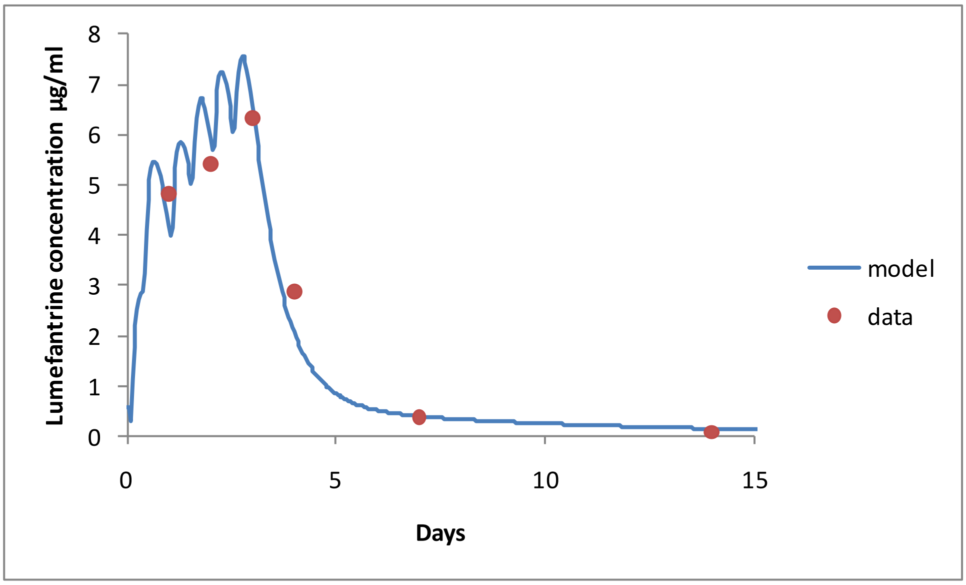

Supplementary Figure 1. Plasma lumefantrine concentrations in African children [1] and model-fitted values. In order to fit an adult PK model [2] to the PK data in children, intercompartmental clearance rates were scaled by bodyweight raised to a constant power: $\mathrm{CL} = \overline{\mathrm{CL}}\left(\frac{\mathrm{W}}{\overline{\mathrm{W}}}\right)^{\gamma}$ where CL is the clearance rate when bodyweight is equal to W kilos, $\overline{\mathrm{CL}}$ is the clearance rate when bodyweight is at its mean value $\overline{\mathrm{W}}$ and γ is a scaling parameter which was estimated at 1.39.

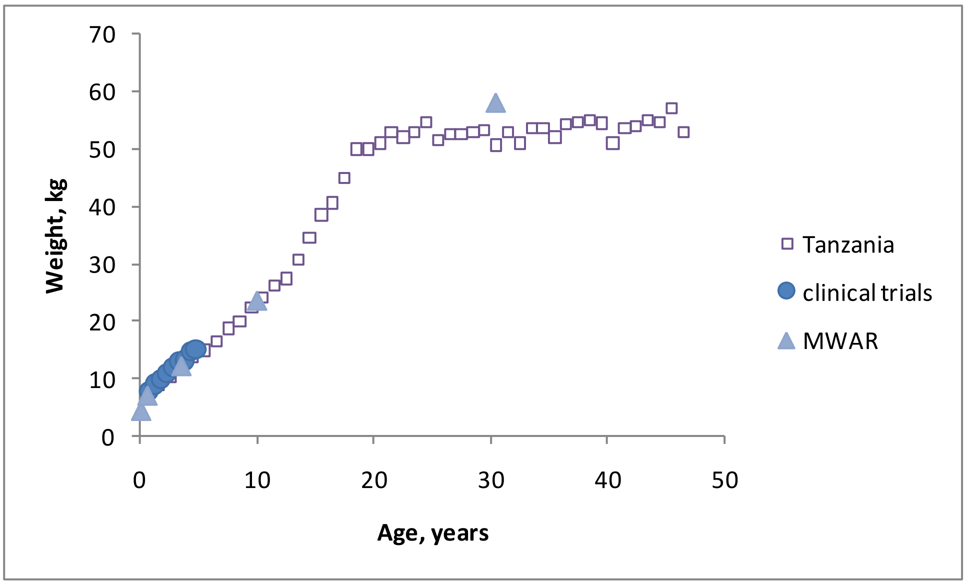

Supplementary Figure 2. Anthropometric data. Age-weight relationships in clinical trials [3,4], the malaria-weighted anthropometric reference (MWAR) dataset [5] and a survey in Tanzania [6].

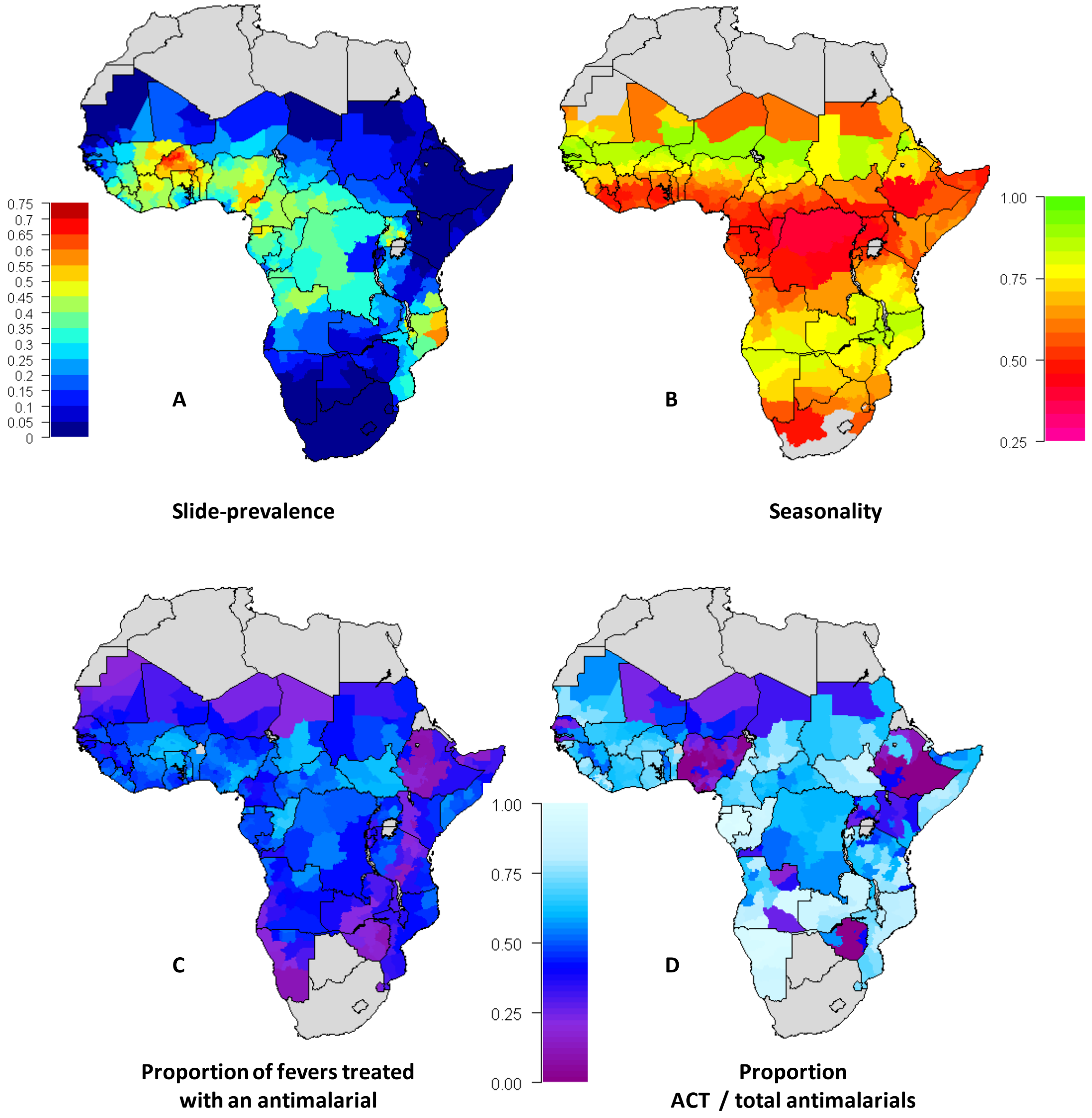

Supplementary Figure 3. Model input data by 1st administrative unit. (A) Slide-prevalence in 2-10 year olds, Malaria Atlas Project 2010 [7]. (B) Summary of seasonal variation in malaria transmission intensity: scale indicates proportion of total annual EIR occurring in the 4 months of the year with highest EIR [8] (daily EIRs are input into the model). (C) Proportion of fevers treated with an antimalarial (D) ACT coverage (proportion of antimalarial intake which is an ACT). (C) & (D) are based on the most recent DHS, MIS or MICS surveys [9] and share the blue-purple scale. In all panels, grey indicates no malaria transmission or no data.

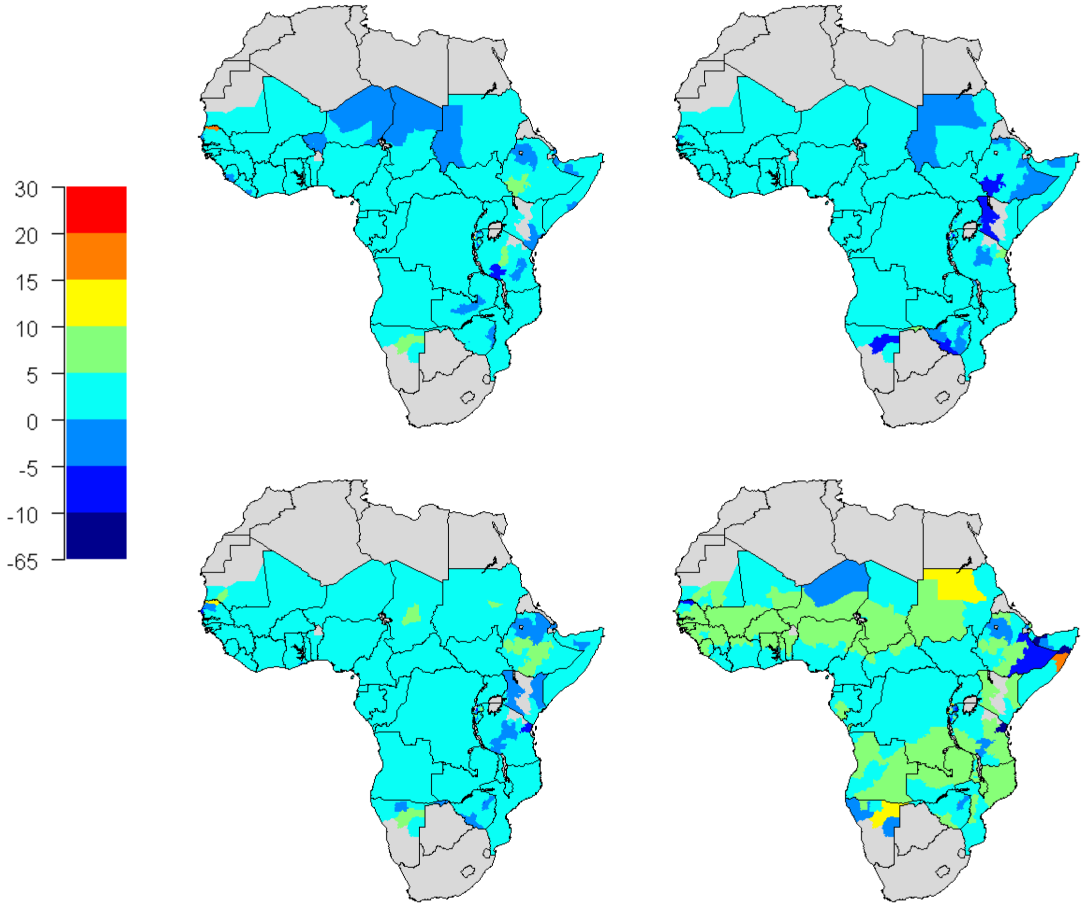

Supplementary Figure 4 Impact of DHA-PQP versus AL on slide-prevalence of infection in all ages by first administrative area: % reduction in the DHA-PQP scenario compared with the AL scenario. Treatment coverage in each of the 4 maps is as described for Figure 4 in the main text.

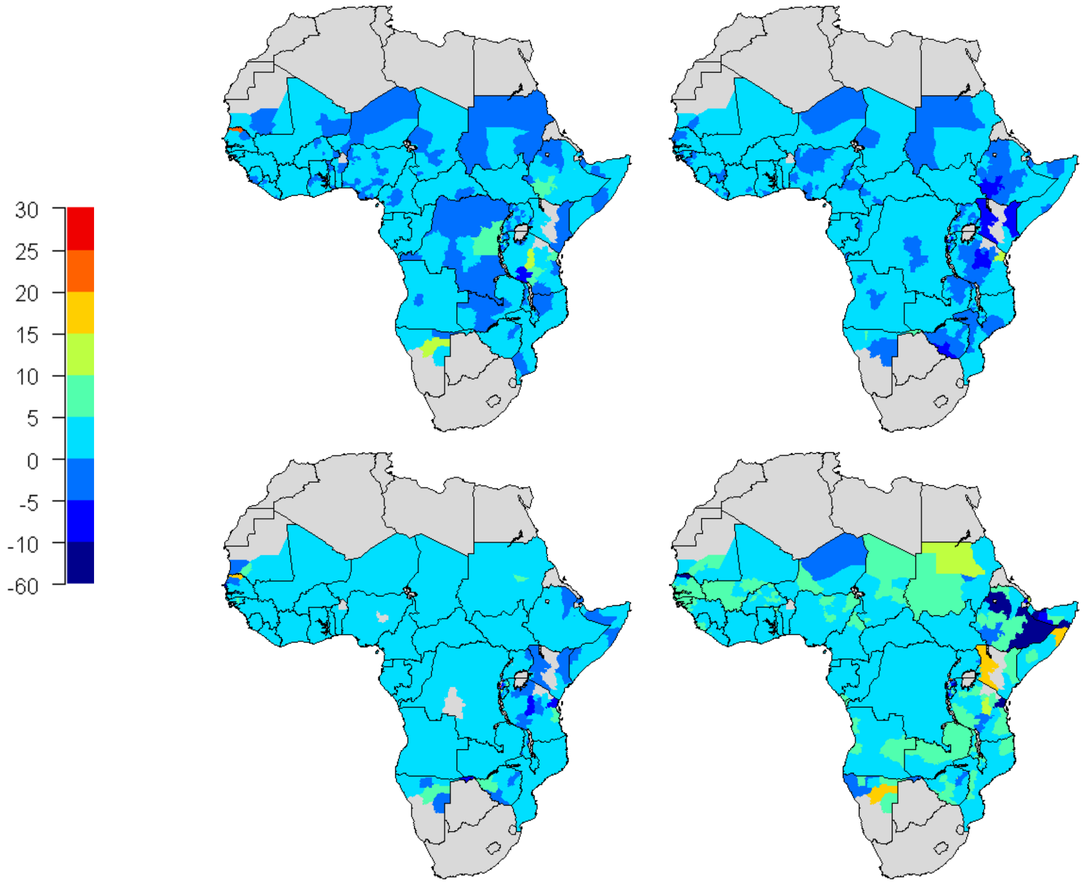

Supplementary Figure 5 Impact of DHA-PQP versus AL on EIR by first administrative region: % reduction in the DHA-PQP scenario compared with the AL scenario. Treatment coverage in each of the 4 maps is as described for Figure 4 in the main text.

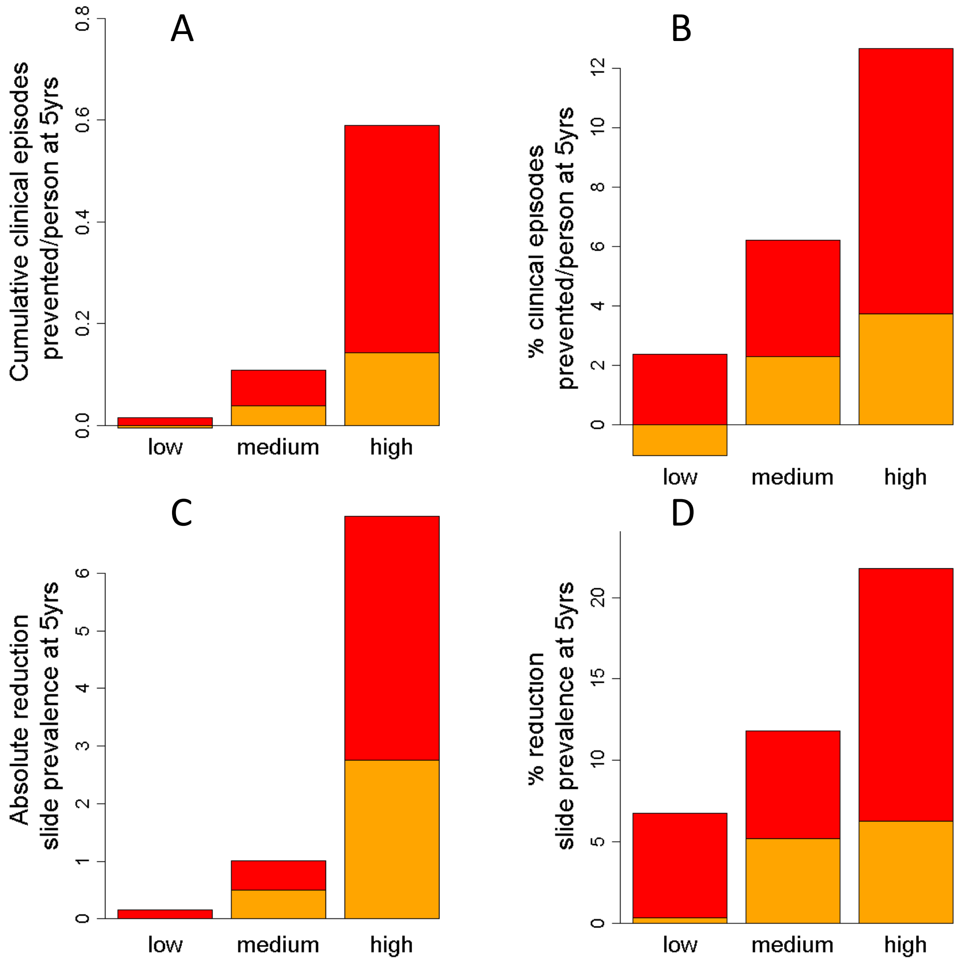

Supplementary Figure 6. Gametocytocidal drug effects: sensitivity analysis**.** Repeat of the analysis shown in Figure 3 in the main text, with lower gametocytocidal effects of treatment (see Table 1, main text). Model-simulated impact on clinical episodes and parasite prevalence in all age groups of having DHA-PQP as first-line treatment versus AL over 5 years in low, medium and high transmission settings with (red) and without (orange) seasonal variation in transmission, assuming high treatment access (80% of cases are treated), but no other interventions. The infectiousness of an individual treated with DHA-PQP here is the same as for an untreated infection (though the duration of treated infection is still assumed to be 5 days). The infectiousness of an AL-treated infection is 1.85-fold lower than for a DHA-PQP-treated infection, i.e. the same effect relative to DHA-PQP as in the main text, but more infectious relative to an untreated infection. Low, medium and high indicate baseline slide-prevalence levels before treatment change of 5, 15 and 50%, respectively, in children aged 2-10 years in the non-seasonal setting. Seasonal settings have the same EIR as the non-seasonal settings. Absolute reductions (A & C) and percentage reductions (B & D) are shown (negative value indicates an increase in cases in the DHA-PQP scenario).

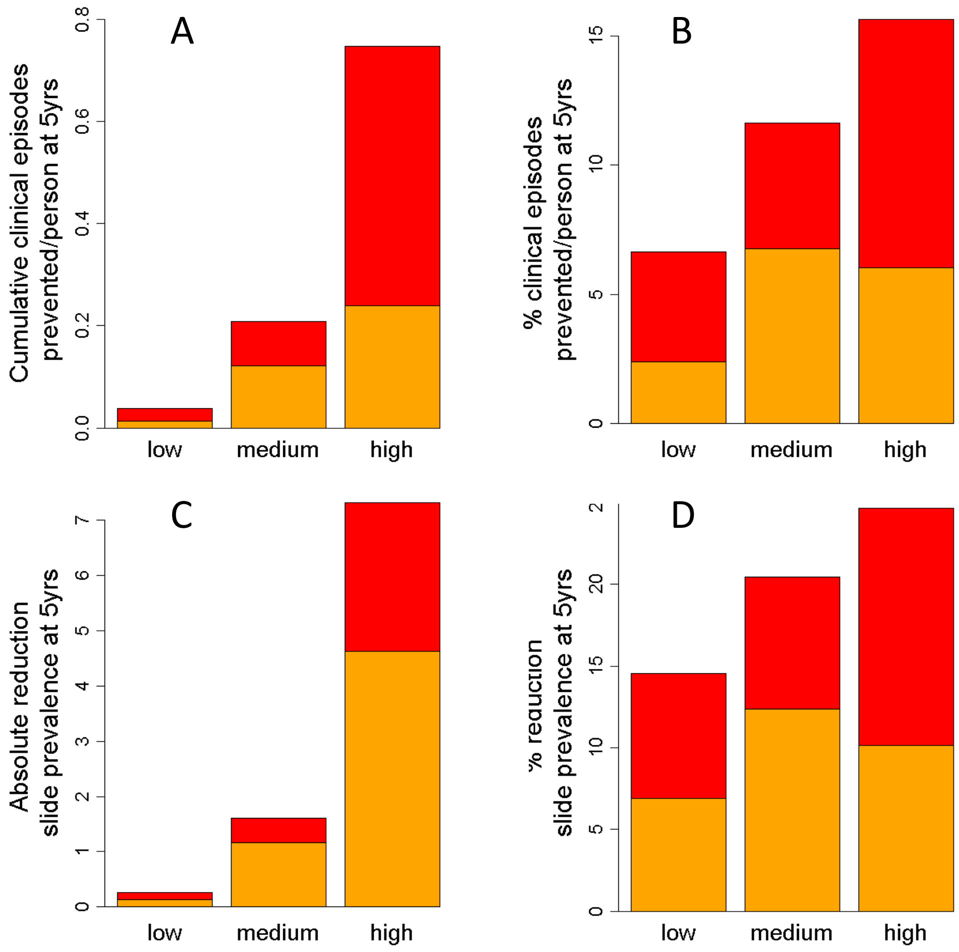

Supplementary Figure 7. Presumptive treatment: sensitivity analysis. Repeat of the analysis shown in Figure 3 in the main text, with the addition of presumptive treatment in asymptomatics and uninfected individuals as well as treatment in symptomatic cases. Model-simulated impact on clinical episodes and parasite prevalence of having DHA-PQP as first-line treatment versus AL over 5 years in low, medium and high transmission settings with (red) and without (orange) seasonal variation in transmission, assuming high treatment access (80% of cases are treated), but no other interventions. Here we make the simple assumption of a constant non-malarial fever rate of 0.75 per person per year [9] in all settings in addition to symptomatic malaria incidence, with any fever also having an 80% chance of receiving the first-line treatment. Low, medium and high indicate baseline slide-prevalence levels before treatment change of 5, 15 and 50%, respectively, in children aged 2-10 years in the non-seasonal simulations. Seasonal settings have the same EIR as the non-seasonal settings. Absolute reductions (A & C) and percentage reductions (B & D) are shown.

Supplementary Table 1. Sensitivity analysis. PKPD & reinfection model parameters with posterior estimates & 95% credible intervals. PKPD parameters were estimated under different sets of model assumptions for sensitivity analysis. Including random effects in the pharmacokinetic model simulations resulted in only small differences in parameter estimates for piperaquine, but a larger difference for lumefantrine, and the analysis which incorporated random effects was therefore selected for transmission simulations (main text). Repeating the analysis without including pre-erythrocytic immunity made little difference to the parameter estimates, but the immunity was included for completeness. Including seasonality in EIR in the analysis made only a small change to parameter estimates and was not included in the final analysis.

**Parameters**	**Symbol**	**Final model in main text**	**Model with no random effects on PK parameters**	**Model with no pre-erythrocytic immunity**	**Including seasonality**	**Units**
Fitted parameters, full PKPD model						
Probability of reinfection as piperaquine concentration tends towards infinity	$P_{\min}$	0.0038 (0.0001-0.0210)	0.0085 (0.0005, 0.0333)	0.0034 (0.0001-0.0181)	-	-
Piperaquine concentration at which the probability of blood stage infection is reduced by half	C_{50}	22.1 (19.9-24.1)	24.3 (22.0, 26.4)	21.6 (19.6-23.8)	-	ng/ml
Piperaquine power parameter	k	21.0 (9.5-40.7)	10.7 (6.5, 20.2)	20.6 (9.6-40.4)	-	-
Probability of reinfection as lumefantrine concentration tends towards infinity	$P_{\min}$	0.0224 (0.0006-0.1017)	0.0542 (0.0058, 0.1552)	0.0217 (0.0008-0.098)	-	-
Lumefantrine concentration at which the probability of blood stage infection is reduced by half	C_{50}	331.0 (229.2-543.4)	429.8 (318.5, 588.6)	302.1 (207.2-480.2)	-	ng/ml
Lumefantrine power parameter	k	12.2 (2.6-34.3)	8.0 (2.2, 30.6)	11.9 (2.4-35.2)	-	-
Annual EIR - Nanoro, Burkina Faso	ε	97.3 (66.2-145.6)	79.3 (52.7, 115.6)	86.4 (57.4-130.1)	-	ibpppy‡
Annual EIR – Kilifi, Kenya	ε	19.6 (11.7-31.6)	17.2 (10.5, 27.2)	19.3 (12-31)	-	ibpppy
Annual EIR - Manhiça, Mozambique	ε	24.9 (16-39.3)	23.1 (15.0, 33.7)	24.3 (16.4-36.2)	-	Ibpppy

Parameters	Symbol	Final model in main text	Model with no random effects on PK parameters	Model with no pre-erythrocytic immunity	Including seasonality	Units
Annual EIR - Mbarara, Uganda	ε	19.8 (11.5-34.3)	17.7 (10.5, 29.3)	18.8 (11.3-30)	-	Ibpppy
Annual EIR - Ndola, Zambia	ε	41.1 (24.7-64.7)	34.3 (21.4, 53.0)	37.8 (23.7-57.9)	-	ibpppy
Annual EIR – Bobo-Dioulasso, Burkina Faso	ε	25.2 (17.1-36.5)	20.9 (14.4, 30.3)	22.4 (15.2-33.1)	-	ibpppy
Fitted parameters: model of prophylaxis without PKPD (Weibull survival curve)						
Piperaquine scale parameter	λ	28.1 (23.6, 34.5)	-	-	30.5 (25.5-36.7)	days
Piperaquine slope parameter	w	4.4 (2.9, 7.6)	-	-	4.3 (3-6.7.0)	-
Lumefantrine scale parameter	λ	10.6 (9.3, 13.1)	-	-	11.9 (9.9-15.2)	days
Lumefantrine slope parameter	w	11.3 (4.0, 32.2)	-	-	7.7 (3.3-27.5)	-
Annual EIR - Nanoro, Burkina Faso	ε	74.4 (48.7-111.5)	-	-	62.4 (40.3-95.6)	ibpppy
Annual EIR – Kilifi, Kenya	ε	17.9 (10.9-28.7)	-	-	15.8 (9.9-25.7)	ibpppy
Annual EIR - Manhiça, Mozambique	ε	21.9 (14.5-33.2)	-	-	16.0 (10.5-24.7)	Ibpppy
Annual EIR - Mbarara, Uganda	ε	16.5 (9.6-27.6)	-	-	14.8 (8.8-24.6)	Ibpppy
Annual EIR - Ndola, Zambia	ε	32.7 (20.6-51.9)	-	-	21.3 (13.3-33.3)	ibpppy
Annual EIR – Bobo-Dioulasso, Burkina Faso	ε	20 (13.6-28.9)	-	-	10.2 (6.9-15.1)	ibpppy

‡ibpppy= infectious bites per person per year

Supplementary methods

Data

Data from two randomized clinical trials of DHA-PQP and AL for treatment of uncomplicated malaria in six different African sites with medium-high transmission intensity (Table 1) were analysed to parameterise a PKPD model. The sites were Nanoro & Bobo-Dioulasso, Burkina Faso; Kilifi, Kenya; Manhiça, Mozambique; Mbarara, Uganda and Ndola, Zambia. [3,4]. Both trials included children aged over six months if they had *P. falciparum* mono-infection with a parasite density of 2,000-200,000 parasites per µl blood. All individuals were tested for parasitaemia on days 1, 2, 3, 7, 14, 21, 28, 35 and 42 after treatment, and on any other day they visited the clinic feeling unwell. DHA-PQP was given once daily for 3 days. The dose of piperaquine per day was 160mg, 320mg, 640mg, and 960mg for the weight bands 7-12kg, 13-23, 24-35, 36-75kg, respectively.

Dosing was similar but not identical in the two trials, with patients in the larger trial receiving 18 mg/kg PQP and 2.25 mg/kg DHA per dose [3], and patients in the smaller trial receiving 17 mg/kg PQP and 2.13 mg/kg DHA per dose [4]. The dosing regimen for AL was identical in both trials, with doses given at 0, 8, 24, 36, 48, and 60 hours. Patients received 1, 2, 3 or 4 tablets (each containing 20 mg artemether and 120 mg lumefantrine) according to the following weight groups: 5-14kg, 15-24kg, 25-34kg, >35kg, respectively. Insecticide-treated nets were given to all patients in the larger trial except those in Kilifi, Kenya, which is taken into account in the analysis (see below).

Since the focus of this analysis was to characterise protection against new infections, we excluded individuals who had recurrent parasitaemia before 14 days of treatment (PCR analysis was not done as these were assumed to be recrudescent infection), those with PCR-confirmed recrudescence after day 14 (n=71), and those who had recurrent parasitaemia on day 14 or later but indeterminate PCR results to distinguish reinfection from recrudescence (n=6). We also excluded individuals who did not follow the trial protocol (n=135), and those with only one non-missing observation (n=2). We discarded observations made after any appearance of recurrent parasitaemia or additional antimalarial treatment (401 observations). We excluded 58 individuals over 10 years old from the smaller trial since the numbers in these age-weight groups were too few to meaningfully estimate the duration of protection. In total, 1,651 individuals with 14,241 observations were included in the analysis of reinfection rates.

PKPD model

The incidence of infection is assumed to be heterogeneous between individuals as in [10]. In brief, exposure to infectious bites is modelled as being log-normally distributed among individuals. We approximate this distribution using Gauss-Hermite quadrature to subdivide each dose-weight group into 3 exposure groups. Age also affects the incidence of infection in two ways: through exposure to bites and pre-erythrocytic immunity [10,11]. Exposure to bites increases with age due to increased body surface area according to the function:

$$\varepsilon(a) = \varepsilon_0 \left(1 - \rho e^{-\frac{a}{a_0}}\right) \qquad (1)$$

where ε_0 is the EIR as measured for adults, $1-\rho$ is the biting rate at birth relative to adults, *a* is the mean age of the dose-weight group and a_0 determines the rate of change of exposure with age. Pre-erythrocytic immunity reduces the probability that inoculated parasites survive to emerge from the liver. Pre-erythrocytic immunity I_{B} in age group *i* and exposure group *j* is acquired with exposure to infectious bites, according to a function identified in previous work which gives the best fit to multiple epidemiological datasets on age prevalence, clinical disease, and vaccine trial data from a range of endemic settings [12]:

$$\frac{dI_{\mathrm{B_{ij}}}}{dt} = \frac{\varepsilon_{\mathrm{ij}}}{\varepsilon_{\mathrm{ij}} u_{\mathrm{b}} + 1} - \frac{I_{\mathrm{B_{ij}}}}{d_{\mathrm{b}}} \qquad (2)$$

where ε is the EIR and u_{b} and d_{b} are constants estimated during previous work which affect the rate of acquisition and loss of pre-erythrocytic immunity [12]. The infection probability *b* in the absence of drug treatment in age group *i* and exposure group *j* is then:

$$b_{\mathrm{ij}} = b_{\mathrm{h}} \left(\frac{1 - b_{\mathrm{min}}}{1 + \left(\frac{I_{\mathrm{B_{ij}}}}{I_{\mathrm{B_0}}} \right)^{q}} + b_{\mathrm{min}} \right) \qquad (3)$$

where b_h is the infection probability in a non-immune individual, b_{min} is the maximum relative reduction in infection probability in a highly immune individual, and I_{B_0} and *q* are constants which influence the midpoint and slope of the immunity function [10].

Reinfection in treated individuals was simulated after the first dose of treatment, assuming a constant force of infection over time. The rate of change in infected individuals in age group *i* and exposure group *j* is:

$$\frac{dI_{\mathrm{ij}}}{dt} = \varepsilon_{\mathrm{ij}} b_{\mathrm{ij}} P (1 - I)_{(t-3.5)} \qquad (4)$$

where I is the cumulative proportion of individuals who have been reinfected, and $(1-I)_{(t-3.5)}$ is the uninfected proportion 3.5 days ago, to allow for the delay in developing slide-positive infection.

Additionally, we explored using a seasonally-varying EIR specific to each of the study sites, based on a previous analysis [8]. Here, rainfall data averaged over a number of years were Fourier-transformed and their relationship with seasonality in clinical malaria incidence characterised. We used the average seasonality pattern for each site, at the resolution of the local first administrative unit. Patient data were subdivided according to their week of recruitment during the year. We assumed a duration of liver stage infection of 2 weeks [13].

Extending PKPD simulations to full populations

We extended pharmacokinetic simulations of piperaquine and lumefantrine concentrations to all age groups as follows. We used the age-weight relationship from a survey to which we had access to high resolution data [6]. These data agreed well with other data from endemic populations in Africa

(Supplementary Figure 2). We categorised individuals into one year age groups up to age 5 years, 2 year age groups up to age 20 and then a single adult age group for those aged over 20 years, since bodyweight plateaus at this age. Pharmacokinetics are age-dependent as well as weight-dependent. We used the models described above to simulate piperaquine pharmacokinetics in children and lumefantrine concentrations in children and adults. To simulate piperaquine concentrations in adults we used a pharmacokinetic model based on non-pregnant women of child-bearing age in Thailand with a mean bodyweight of 48kg (range 37-78kg) [14]. The adult pharmacokinetic models were used for age groups with a mean bodyweight of >36kg. The estimated pharmacodynamic relationships were assumed constant across age groups and used to generate profiles of the reduced probability of reinfection over time after treatment in each age group, which were input into the full transmission model.

Supplementary References

1. Djimde AA, Tekete M, Abdulla S, Lyimo J, Bassat Q, et al. (2011) Pharmacokinetic and pharmacodynamic characteristics of a new pediatric formulation of artemether-lumefantrine in African children with uncomplicated Plasmodium falciparum malaria. Antimicrob Agents Chemother 55: 3994-3999.
2. Tarning J, McGready R, Lindegardh N, Ashley EA, Pimanpanarak M, et al. (2009) Population pharmacokinetics of lumefantrine in pregnant women treated with artemether-lumefantrine for uncomplicated Plasmodium falciparum malaria. Antimicrob Agents Chemother 53: 3837-3846.
3. Bassat Q, Mulenga M, Tinto H, Piola P, Borrmann S, et al. (2009) Dihydroartemisinin-piperaquine and artemether-lumefantrine for treating uncomplicated malaria in African children: a randomised, non-inferiority trial. PLoS One 4: e7871.
4. Zongo I, Dorsey G, Rouamba N, Dokomajilar C, Sere Y, et al. (2007) Randomized comparison of amodiaquine plus sulfadoxine-pyrimethamine, artemether-lumefantrine, and dihydroartemisinin-piperaquine for the treatment of uncomplicated Plasmodium falciparum malaria in Burkina Faso. Clin Infect Dis 45: 1453-1461.
5. Taylor WR, Terlouw DJ, Olliaro PL, White NJ, Brasseur P, et al. (2006) Use of weight-for-age-data to optimize tablet strength and dosing regimens for a new fixed-dose artesunate-amodiaquine combination for treating falciparum malaria. Bull World Health Organ 84: 956-964.
6. Drakeley CJ, Corran PH, Coleman PG, Tongren JE, McDonald SL, et al. (2005) Estimating medium- and long-term trends in malaria transmission by using serological markers of malaria exposure. Proc Natl Acad Sci U S A 102: 5108-5113.
7. Gething PW, Patil AP, Smith DL, Guerra CA, Elyazar IR, et al. (2011) A new world malaria map: Plasmodium falciparum endemicity in 2010. Malar J 10: 378.
8. Cairns M, Roca-Feltrer A, Garske T, Wilson AL, Diallo D, et al. (2012) Estimating the potential public health impact of seasonal malaria chemoprevention in African children. Nat Commun 3: 881.
9. Cohen JM, Woolsey AM, Sabot OJ, Gething PW, Tatem AJ, et al. (2012) Public health. Optimizing investments in malaria treatment and diagnosis. Science 338: 612-614.
10. Griffin JT, Hollingsworth TD, Okell LC, Churcher TS, White M, et al. (2010) Reducing Plasmodium falciparum malaria transmission in Africa: a model-based evaluation of intervention strategies. PLoS Med 7: e1000324.
11. Port GR, Boreham PFL, Bryan JH (1980) The relationship of host size to feeding by mosquitoes of the Anopheles gambiae Giles complex (Diptera: Culicidae). Bull Entomological Res 70.
12. Griffin JT, Ferguson NM, Ghani AC (2014) Estimates of the changing age-burden of Plasmodium falciparum malaria disease in sub-Saharan Africa. Nat Commun 5: 3136.
13. Eyles DE, Young MD (1951) The duration of untreated or inadequately treated Plasmodium falciparum infections in the human host. J Natl Malar Soc 10: 327-336.
14. Tarning J, Rijken MJ, McGready R, Phyo AP, Hanpithakpong W, et al. (2012) Population pharmacokinetics of dihydroartemisinin and piperaquine in pregnant and nonpregnant women with uncomplicated malaria. Antimicrob Agents Chemother 56: 1997-2007.
